# Equity monitoring for social marketing: use of wealth quintiles and the concentration index for decision making in HIV prevention, family planning, and malaria programs

**DOI:** 10.1186/1471-2458-13-S2-S6

**Published:** 2013-06-17

**Authors:** Nirali M Chakraborty, Rebecca Firestone, Nicole Bellows

**Affiliations:** 1 Population Services International, Washington DC, USA; 2 Independent Data Analyst Consultant, Nairobi, Kenya

## Abstract

**Background:**

The majority of social marketing programs are intended to reach the poor. It is therefore essential that social marketing organizations monitor the health equity of their programs and improve targeting when the poor are not being reached. Current measurement approaches are often insufficient for decision making because they fail to show a program's ability to reach the poor and demonstrate progress over time. Further, effective program equity metrics should be benchmarked against a national reference population and consider exposure, not just health outcomes, to measure direct results of implementation. This study compares two measures of health equity, concentration indices and wealth quintiles, using a defined reference population, and considers benefits of both measures together to inform programmatic decision making.

**Methods:**

Three datasets from recent cross-sectional behavioral surveys on malaria, HIV, and family planning from Nepal and Burkina Faso were used to calculate concentration indices and wealth quintiles. Each sample was standardized to national wealth distributions based on recent Demographic and Health Surveys. Wealth quintiles were generated and concentration indices calculated for health outcomes and program exposure in each sample. Chi-square and t-tests were used to assess statistical significance of results.

**Results:**

Reporting wealth quintiles showed that recipients of Population Services International (PSI) interventions were wealthier than national populations. Both measures indicated that desirable health outcomes were usually concentrated among wealthier populations. Positive and significant concentration indices in all three surveys indicated that wealth and program exposure were correlated; however this relationship was not necessarily linear. In analyzing the equity of modern contraceptive use stratified by exposure to family planning messages in Nepal, the outcome was equitable (concentration index = 0.006, p = 0.68) among the exposed, while the wealthy were more likely to use modern contraceptives (concentration index = 0.071, p < 0.01) among the unexposed.

**Conclusions:**

Using wealth quintiles and concentration indices together for equity monitoring improves usability of findings for decision making. Applying both metrics, and analyzing equity of exposure along with health outcomes, provides results that have statistical and programmatic significance. Benchmarking equity data against national data improves generalizability. This approach benefits social marketers and global health implementers to improve strategic decision making and programs' ability to reach the poor.

## Background

The majority of social marketing programs are intended to reach the poor, but like other global health programs, they run the risk of reaching the better educated and higher income segments of a population, who are likely to be healthier than their more disadvantaged counterparts [1]. As a result, programmatic impact may be limited and programs risk reinforcing social and economic inequalities. For this reason, many public health program implementers operating in low and middle-income countries (LMICs) aim to not only generate positive health impact, but to also improve the distribution of health benefits by targeting the most vulnerable and disadvantaged [2].

In the past decade, an improved ability to identify and monitor health equity has allowed implementers, donors, and other global health stakeholders to focus more attention on the issue. Health equity is defined as 'the absence of unfair and avoidable or remediable differences in health among population groups defined socially, economically, demographically or geographically' [3,4]. Equity underpins the health-related Millennium Development Goals (MDGs), with progress towards these goals monitored in terms of equity impact. In addition, and as a likely result of this global focus on equity, the global health equity evidence base has expanded considerably [5-10].

Monitoring health equity is particularly important for social marketing organizations. Social marketing employs marketing techniques to achieve public health goals, reaching populations in need with health products and services that range from free to heavily subsidized in price [11]. Social marketers typically target the poorest segments of the population with fully subsidized (free) products and services. They market partially subsidized products and services and charge a modest price to segments of the population with some means, leaving wealthier segments of the population to be served by the commercial sector. Increasingly, social marketing organizations are using a "total market approach" (TMA) to ensure these population segments are served in this manner. Using a TMA approach also challenges social marketers to meet the needs of the poor and vulnerable in a cost-effective and efficient manner [12-14]. Given these priorities, health equity has major implications for decision making and for assessing the impact of programs.

### Common health equity measures

The most common measures used for equity monitoring are wealth quintiles and the concentration index. Wealth quintiles rank the cumulative distribution of any population-based measure of health or well-being by a measure of socioeconomic status (SES), dividing the population into five groups that represent an equal 20% of the population, ranging from the group that represents the poorest 20% of the population up to those in the wealthiest 20%. By convention, quintile 1 is the poorest segment of the population and quintile 5 is the wealthiest. Global health researchers, implementers, and policymakers have examined health outcomes by wealth quintiles in order to monitor progress towards the MDGs [15], in the Demographic and Health Surveys [5,16] as well as a range of independent studies [17,18]. Combining health outcomes with wealth quintiles shows whether and how outcomes are concentrated in different socioeconomic groups. As a result, researchers and implementers can gain insight into how interventions are reaching each quintile in order to improve targeting in future interventions. Wealth quintiles also demonstrate a country or a program's achievements in health equity compared to those of other populations, and make no assumption about the shape of the relationship between socioeconomic status and health status.

Another commonly used measure to assess equity is the concentration index, which uses one summary value to capture the magnitude of socioeconomic inequality in a health outcome. The concentration index ranges from -1 to 1, based on a Lorenz concentration curve that orders the population by SES on the *x-axis *and plots the cumulative percentage of a health outcome on the *y-axis*. With zero signifying perfect equality, a negative value represents the health outcome's concentration among the poor; a positive value denotes concentration among the wealthy. As the concentration index moves further away from zero, either positively or negatively, there is greater inequity in the health outcome [19]. The concentration index offers advantages as a metric of health equity because it is statistically comparable across time periods and geographic regions.

Both wealth quintiles and the concentration index can be calculated using any measure of socioeconomic status that allows the population of interest to be ranked from highest to lowest by SES. One of the major advances in the past decade for health equity studies in LMICs is the development of an asset index calculated using principal components analysis (PCA), generally regarded as a valid, reliable, and easily interpreted method of measuring household wealth [16,20,21]. This method creates an asset index by ranking households, usually within a nationally representative sample, via a list of household material assets. Each household is given a specific asset score. These asset index scores can then be ranked to distinguish the relatively wealthy from the relatively poor. Publicly available data from DHS surveys, which are nationally representative by design and available in many LMICs, now routinely include information on assets owned by a household.

### Measuring health equity at PSI

Population Services International (PSI) is a global health implementer that works in LMICs around the world to improve the health of poor and vulnerable people, principally through the social marketing of health products and services [22]. Social marketing engages private sector resources and uses private sector techniques to encourage healthy behavior and make markets work for the poor. It is critical for PSI's success to be able to assess whether: 1) the organization improves equity in the health behaviors that it aims to influence; and 2) interventions are actually reaching the poor and most vulnerable as part of programmatic targeting. PSI's measurement program has sought to develop and implement a set of metrics that can be used to regularly monitor the organization's health equity goals.

Given PSI's scope of work and its commitment to demonstrating progress in its programs over time, PSI implementers need a measure that accounts for different time frames and geographies. Since 2007, PSI has used the concentration index as its health equity metric because it produces a summary measure that is easily comparable across programs, countries, and time periods. PSI's Research and Metrics department regularly calculates concentration indices by country for a set of key health outcomes. To do so, it uses data from cross-sectional surveys that are designed to monitor and evaluate PSI programs. Health outcomes may include product or service use as well as the practice of particular behaviors; PSI does not routinely measure clinical outcomes or mortality. The organization uses a PCA-based asset index as the SES measure in calculations. In cases where surveys have not collected asset information, education is used as a proxy [23].

While this approach assesses change over time in health equity, there are several limitations to PSI's current method. Asset indices are calculated from within the study sample, which is generally representative of the specific locations where PSI operates, but usually not of a country's entire national population. As such, making comparisons to a national distribution of socioeconomic status is impossible. Moreover, there is a risk of misclassification bias such that some households may appear rich within the sample, yet actually be relatively poor on a national level. Local research teams have typically used local conditions to set the lists of assets included in questionnaires, so the content of these asset indices is highly variable. Consequently, comparability of results with other surveys and the ability to generalize conclusions to other populations is significantly reduced.

Another limitation to PSI's use of the concentration index is its interpretation. Currently, PSI provides guidance on how to interpret general trends in the concentration index, such that a movement in the index's value towards zero, perfect equity, is considered favorable. In addition, the calculation of standard errors for the concentration index enables researchers and implementers to assess whether differences between concentration indices are statistically significant. However, no guidance exists on what may constitute programmatic significance, the threshold of inequity that merits changes in a program or policy. As a result, it is challenging for implementers to determine when concentration indices are actionable and when better targeting of the poor may be needed.

Finally, PSI currently only looks at equity in health outcomes. With a few exceptions, the current approach does not consider equity in relation to exposure to its interventions. This is a shortcoming of the approach, especially since exposure measures reflect the direct results of PSI's work in product distribution, service delivery, or behavior change communications (BCC). To fully understand the extent of health equity in programming, equity in exposure should be measured along with equity in health outcomes.

### Study goals

We use survey data to compare the merits of using concentration indices and wealth quintiles to measure equity among social marketing program recipients. We also consider the benefit of using both measures together to inform programmatic decision making. For a measure or set of measures to be useful for programmatic decision making, the results should be easy to interpret, as precise as possible, and representative of the populations under study. Our goal is to develop a method that social marketers and other implementers in global health can use for equity monitoring. Doing so will help implementers understand the effectiveness of their programs and their ability to reach the poor.

## Methods

### Data sources

We used data from three recent cross-sectional behavioral surveys, representing three key health areas in which PSI works: malaria, HIV, and family planning (Table 1). To identify eligible datasets, we used the following criteria: 1) existence of a recent national DHS survey in the same country, with asset and dwelling space variables comparable to those in the PSI datasets (see Additional file 1 for common variables between the datasets); 2) a clearly defined outcome used to measure program success; and 3) several variables indicating exposure to a PSI intervention. When determining inclusion criteria, we did not set a criterion that PSI studies have comparably measured outcomes to the DHS; in only one survey (Nepal family planning), the same outcome (modern contraceptive use) was measured in both the PSI survey and the DHS. All of the PSI surveys considered for this study received approval for human subjects protection by either the PSI Research Ethics Board or a local Institutional Review Board.

**Table 1:** Description of datasets and reference populations

	Malaria Survey	HIV Survey	Family Planning Survey
**Country**	Nepal	Burkina Faso	Nepal

**Year**	2010	2010	2011

**Total number of households **	3,327	730	1,078

**Population under analysis**	Pregnant women (n = 195) Children under 5 (n = 1,805)	Youth, aged 15-24 (n = 568)	Non-pregnant married women, aged 18-49 (n = 1,036)

**Key outcome variables**	% slept under any bednet last night % slept under LLIN last night	% condom use at last sex % consistent condom use	% using modern contraceptives

**Key exposure variables**	% saw a Supanet poster % received home visit	% saw any of 3 PSI ads	% heard any intrauterine device (IUD) message % saw branded IUD poster/leaflet

**Reference population**	Nepal DHS, 2010 (n = 10,826)	Burkina Faso DHS, 2003 (n = 9,097)	Nepal DHS, 2010 (n = 10,826)

The 2010 Nepal malaria survey took place during the second year of a three-year campaign promoting long-lasting, insecticide-treated bednets (LLINs) in 13 districts where malaria is endemic (unpublished data, PSI, 2010). The sample was stratified by phases of the campaign, such that there were three strata, each representing one phase of the communications and net distribution campaign. Within each stratum, clusters (wards or villages) were sampled using probability proportional to size (PPS) sampling. Stratum-specific weights were applied in the analysis. Households within clusters were listed, and eligible respondents, caregivers of children under the age of five, were randomly sampled from within the household. The DHS in Nepal was conducted in 2010 and is nationally representative [24].

The PSI family planning survey in Nepal took place in 2011, during the third year of a multi-year reproductive health program aimed at increasing the use of long-acting contraceptive methods and medication abortion in 47 of the country's 75 districts (unpublished data, PSI, 2011). Twenty-three of 47 program districts were selected using PPS. Within selected districts, clusters (wards or villages) were systematically selected, also based on PPS. As with the Nepal malaria survey, the researchers used systematic random sampling from the households within each cluster to find eligible respondents, who were married women between 15 and 49 years old. This dataset was also compared to the Nepal 2010 DHS sample [24].

For the final PSI dataset, we chose the 2010 Burkina Faso HIV survey, a cross-sectional survey implemented in the final year of a four-year HIV prevention and family planning program [25]. The survey is nationally representative, and the sample was proportionately distributed between urban and rural clusters, with clusters within each strata selected via PPS, resulting in a self-weighted sample at the household level. Simple random sampling within the cluster was used to select two samples: (1) youth (aged 15-24 years) and (2) adults (aged 25-49 years), both from households in the cluster. Within these two groups, the survey restricted eligibility to individuals who had had sex with a non-marital, non-cohabiting partner in the last 12 months. Due to the low proportion of adults who met this criterion, analyses in this paper were restricted to youth aged 15-24 years. The corresponding DHS in Burkina Faso was conducted in 2003 and is nationally representative [26].

### Analytic methods

#### 1. Standardization of PSI samples to national distribution of wealth

For each of the three datasets described above, identical methods were followed. In order to analyze equity in health outcomes and exposure to PSI programs, the survey samples were first placed within the national distribution of household wealth. The first step required identifying the asset and household variables common to both the PSI survey and the corresponding DHS survey. A total of 29 binary variables in the Nepal datasets and 12 binary variables in the Burkina Faso dataset met this criterion (see Additional file 1).

Next, using the common variables and the DHS datasets, we conducted principal components analysis to generate an asset index for each country [21]. To calculate the asset score for each household in the DHS sample (A_i1_), PCA sums the standardized value of each variable multiplied by its eigenvalue, such that $\mu_{\hat{v}}=0$ and $\sigma_{\hat{v}}=1$ (the mean and standard deviation of $\hat{v}=0,1$) and ${\hat{v}}_{i}$ is multiplied by the eigenvalue (ε_v_) of the first principal component for that variable (Equation 1) [27].

(1) $$A_{i1}=\sum_{1}^{v}\varepsilon_{v}\times {\hat{v}}_{i}$$

For a sensitivity analysis of the reduced set of variables included in the PCA, we estimated the correlation of the asset scores generated from the full set of asset and household variables available in the DHS. Correlations between these two indices from the DHS data were extremely high (ρ ≥ 0.99).

After calculating the asset scores with the variables common to the PSI and DHS surveys for each country, we ranked the resulting asset scores for each household in the DHS dataset from lowest to highest. Then, we divided the DHS data into quintiles based on their asset score, with approximately 20% of the population in each quintile. The cut-off values for the quintiles, demarcating the upper and lower limits of each quintile, were retained.

Next, we created an asset score (A_i2_) for the households in each of the three PSI surveys, standardizing each variable (v_i2_) against the DHS distribution $\left(\mu_{\hat{v}},\sigma_{\hat{v}}\right)$, and multiplying these variables by the DHS eigenvalue (ε_v_) (Equation 2).

(2) $$A_{i2}=\sum_{1}^{v}\left(\varepsilon_{v}\times \frac{v_{i2}-\mu_{v}}{\sigma_{v}}\right).$$

This process of multiplying standardized values by the factor scores from the national distribution placed the wealth of households in the PSI sample within the DHS-based national wealth distribution of the country of interest. Doing so allowed for wealth comparisons within the PSI sample and created a benchmark to the national distribution of wealth.

#### 2. Generate wealth quintiles for PSI samples

The second step was to assign each household in the PSI samples to a wealth quintile. To do so, we classified each of the PSI household asset scores into a group (Q1 through Q5), according to the cut-off values retained from the DHS wealth quintiles.

#### 3. Calculation of concentration index

For individual level data, we calculated the concentration index from the concentration curve, which was generated by ranking the population by asset score on the *x-axis *and plotting the cumulative percentage of the outcome variable of interest on the *y-axis*. This calculation was achieved using the STATA command GLCURVE [28]. The concentration index, then, is equal to twice the area between the curve and the line of equality (x = y), or 2cov(y_i_,x_i_)/μ_y_, where x_i _is the fractional rank of the i^th ^individual [28].

We calculated concentration indices and quintile-specific proportions for the following health outcomes: use of any bednet; use of an LLIN; use of modern contraceptives; condom use at last sex by partner type; and consistent condom use by partner type. We also calculated concentration indices and quintile-specific proportions for measures of the proportion of the population exposed to a PSI message or interventions. These exposure variables included: saw a PSI-branded poster on LLIN promotion; received a home visit for LLINs; saw any of three PSI HIV prevention advertisements; heard any IUD message; and saw a PSI-branded IUD leaflet or poster. Finally, we calculated quintile-specific proportions and concentration indices for the health outcome variables after stratifying by exposure status.

### Significance testing

For the wealth quintiles, we conducted a χ^2 ^test of equality of proportions to test for significant differences between quintiles in the proportion of the population with each outcome. We also calculated the bounds of 95% confidence intervals for quintile-specific estimates. We calculated standard errors for each concentration index, testing whether each concentration index was statistically different from zero. For concentration indices of outcomes calculated for exposed and unexposed groups, we also used a t-test of differences in means to test whether these estimates were significantly different from each other. STATA 11 (StataCorp LP) was used for all statistical analyses.

## Results

**Figure 1:**
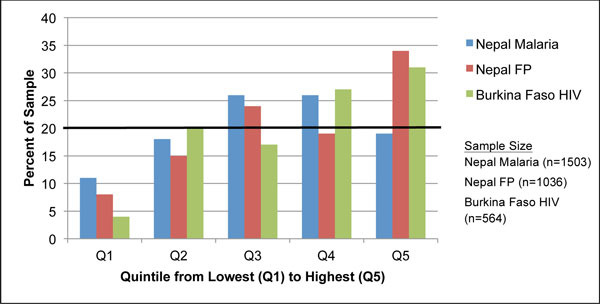
**Distributions of survey respondents by wealth quintile and survey**. The black horizontal line at 20% denotes the cut-off level for each quintile if wealth were distributed with perfect equity in the study population. Quintile distribution of the reference populations, the corresponding DHS dataset for each PSI survey, is evenly distributed; all quintiles represent 20% of the sample.

**Figure 2:**
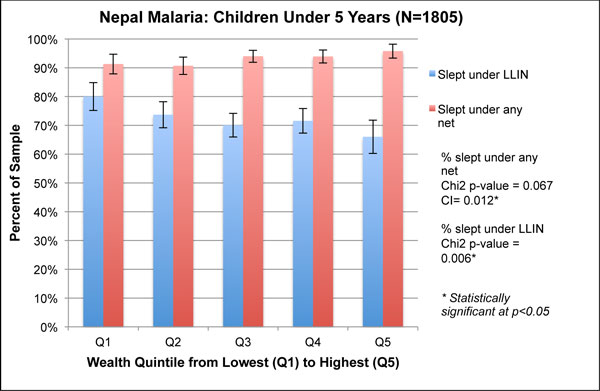
**Wealth quintiles and concentration indices for malaria-related outcomes of children under five in Nepal, 2010**.

**Figure 3:**
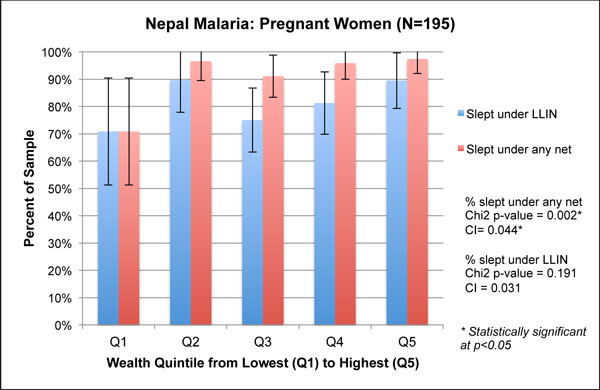
**Wealth quintiles and concentration indices for malaria-related outcomes of pregnant women in Nepal, 2010**.

**Figure 4:**
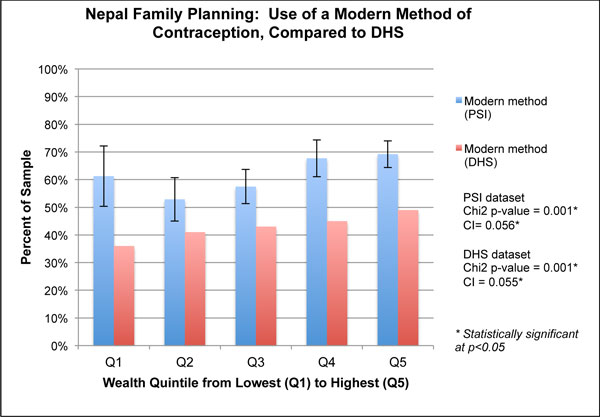
**Wealth quintiles and concentration indices for family planning use in Nepal, 2011**.

**Figure 5:**
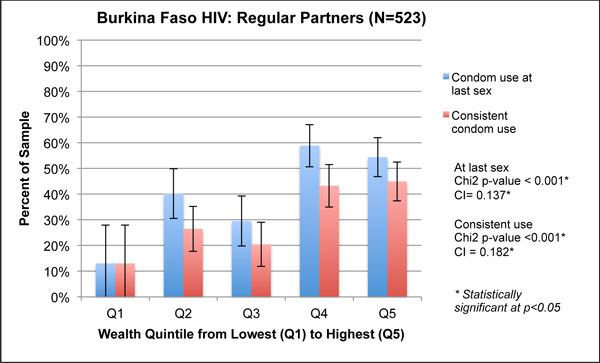
**Wealth quintiles and concentration indices for HIV-related outcomes among regular partners in Burkina Faso, 2010**.

**Figure 6:**
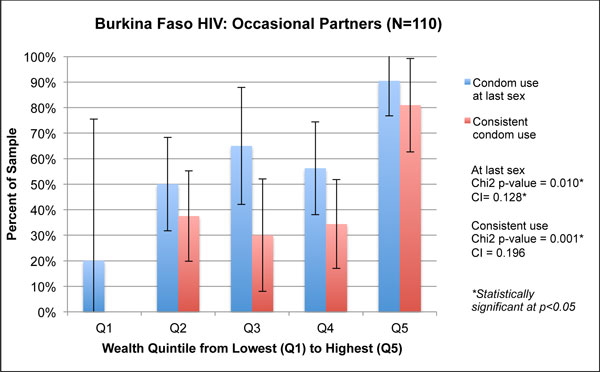
**Wealth quintiles and concentration indices for HIV-related outcomes among occasional partners in Burkina Faso, 2010**.

**Figure 7:**
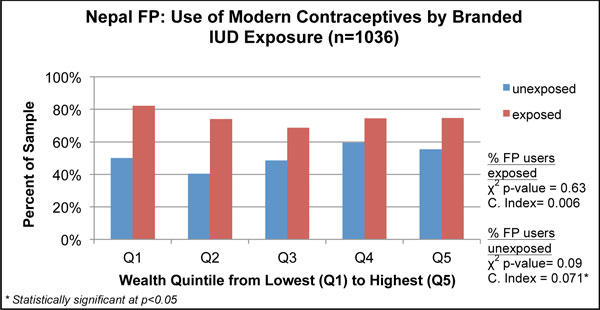
**Wealth distributions for health outcomes, stratified by exposure to PSI's interventions**.

Tables 2 and 3 and Figures 1 - 7 present the results of this analysis. Table 2 presents descriptive statistics for each of the three PSI samples. The Nepal malaria survey consisted of predominately married men and women averaging 26 years in age, while the Nepal family planning survey focused exclusively on married women with a higher average age, 30 years. In contrast, Burkina Faso's survey focused on a younger (average age 20 years) and mostly single population. The education variables for all three surveys showed that approximately 40% of the samples had no education while another 40% had either attended some or completed secondary school; the remainder had either some or completed primary school (Table 2).

**Table 2:** Descriptive characteristics of respondents in PSI surveys

	Nepal Malaria Survey, 2010 (n = 1,503)	Nepal Family Planning Survey, 2011 (n = 1,036)	Burkina Faso HIV Survey, 2010 (n = 568)
**Gender**			
Respondent*			
Male	54%	-	59%
Female	46%	100%	41%

**Average age of respondent **(yrs)	26	30	20

**Marital status of respondent**			
Single (never married)	0%	-	92%
Married/in-Union	>99%	100%	7%
Separated/Divorced/Widowed	<1%	-	1%

**Education of respondent**			
None	42%	44%	41%
Some or completed primary	15%	14%	21%
Some or completed secondary	43%	42%	38%

**Employment status**			
Employed	56%	33%	51%
Student	1%	<1%	33%
Unemployed/Homemaker	43%	67%	17%

*In the Nepal malaria survey, gender refers to children under five years of age. The survey respondents for these children were all women.

Figure 1 depicts the wealth distribution of the three survey samples ranked from poorest quintile (Q1) to wealthiest (Q5), using the national DHS reference population. Overall, the PSI samples were wealthier than the DHS populations, as seen in Figure 1, indicating that greater than 20% of the PSI sample respondents fell within the fourth and fifth quintiles. In the Nepal malaria survey, over 10% of the sample was represented in each quintile, with the largest cluster of observations in the middle-income quintile (Q3) (26%). In contrast, the Nepal family planning and Burkina Faso HIV surveys reported fewer observations in the poorest quintile (8% for Nepal and 4% for Burkina Faso) and the most observations in the wealthiest quintile (34% for Nepal and 31% for Burkina Faso). More than half of the observations in the Burkina Faso survey were concentrated in the wealthiest quintiles (Q4 and Q5).

### Equity of health outcomes

Figures 2 through 6 show the relationship between wealth and the key health outcomes for each survey. Further details on this analysis are presented in Additional file 2. The bar charts depict results stratified by wealth quintile with the results of the χ^2 ^test indicating if any difference detected in the proportions across the quintiles is statistically significant. The 95% confidence interval of each quintile-specific proportion is also shown as brackets in Figures 2 through 6. The concentration index provides an overall summary measure of equity for each outcome, and was tested to determine if the results were significantly different from zero, indicating that inequity was present.

In the Nepal malaria survey, households reported that the vast majority of children under five slept under any bednet, ranging from 90% in Q1 to 95% in Q5 (Figure 2). While no significant difference was found between the quintiles (χ^2 ^p = 0.067), the concentration index of 0.012 was statistically significant (p < 0.01), indicating concentration of bednet use among the wealthiest. By contrast, poorer children were significantly more likely to have slept under an LLIN than the richest children, which is demonstrated by a concentration index of -0.035 (p < 0.01) and significantly higher levels of use among the poorer quintiles in the graph (χ^2 ^p = 0.006).

For pregnant women, sleeping under any bednet was significantly associated with greater wealth (concentration index = 0.044, p < 0.01; χ^2 ^p = 0.002). In contrast, sleeping under an LLIN was not associated with wealth (concentration index = 0.031, p = 0.17; χ^2 ^p = 0.191) (Figure 3). The graphical analysis indicates that use of an LLIN was non-linear and higher in Q2 (87%) than in Q3 or Q4, although these differences are within the confidence intervals. This finding is likely due to the small sample of pregnant women in this survey.

The Nepal family planning analysis shown in Figure 4 considers the proportion of women using a modern method of contraception and compares contraceptive use by SES among the study population of the PSI program to the national reference population in the DHS. For both study populations, modern contraceptive use was higher in wealthier populations. The concentration index was 0.056 (p < 0.01) for the PSI survey and 0.055 (p < 0.01) in the DHS, though the DHS national estimate of modern family planning use was lower. Looking at the wealth quintiles, both samples had significant differences across the quintiles (χ^2 ^p = 0.001). Unlike the DHS results that showed a gradual, but steady, increase in the use of modern contraceptives from Q1 to Q5, the PSI data showed a substantial dip from Q1 to Q2, followed by an increase to the wealthiest strata (Q4 and Q5). Differences between adjacent quintiles appear within the boundaries of the confidence intervals.

Figures 5 and 6 show a strong association between wealth and health outcomes in the Burkina Faso HIV survey. Both condom use at last sex and consistent condom use, for regular partners and occasional partners, showed significantly different distributions by quintile based on the χ^2 ^test. Few respondents were concentrated in Q1 for either outcome, which explains the wide confidence intervals in Figures 5 and 6 for this quintile. For condom use with occasional partners, we note a large spike in Q5: 90% of respondents in Q5 reported condom use at last sex with occasional partners, a difference of 20% from those in Q1 who reported the same. Similarly, 81% of respondents in Q5 reported consistent condom use with occasional partners, compared with 0% reporting this outcome in Q1. For both types of condom use, all four concentration indices were positive; three of the four were statistically significant. The concentration index for condom use at last sex with regular partners was 0.137 (p < 0.01), and only slightly lower with occasional partners, 0.128 (p = 0.04).

### Equity of intervention exposure

Table 3 details equity results related to exposure to PSI interventions. Program exposure ranged from viewing or hearing BCC messages via mass media or printed materials (leaflets or posters), to learning about the health behavior through interpersonal communication with an outreach worker (e.g., home visits). Overall, we saw a relatively steady increase in exposure from Q1 to Q5 and positive concentration indices in each survey. This indicates that the wealthier were more exposed to PSI's social marketing campaigns than poorer individuals. The exposure measure used in the Burkina Faso survey (exposed to at least one of three advertisements) was the only one that did not show a steady increase in campaign exposure from Q1 to Q5. Instead, exposure increased from Q1 to Q2, decreased in Q3, and rose again in Q4 and Q5. The concentration index of 0.236 (p < 0.01) for this measure from Burkina Faso was the highest of the exposure concentration indices, indicating that this variable had the highest concentration among the wealthy.

**Table 3:** Wealth quintiles and concentration indices for exposure to PSI interventions

Exposure	N	Overall	Q1	Q2	Q3	Q4	Q5	χ^2^	C. Index	SE_C.Index_
*Nepal Malaria Survey^a^*										

% saw PSI-branded leaflet or poster	1,503	64	50	59	59	71	78	<0.001*	0.085*	0.011

% received an at home visit regarding LLIN	1,503	28	23	26	27	31	30	0.221	0.056*	0.024

*Nepal Family Planning Survey *										

% heard or saw any IUD health message	818^b^	41	29	31	38	38	48	0.004**	0.096*	0.024

% saw PSI-branded IUD leaflet or poster	1,036^c^	54	35	37	44	54	71	<0.001**	0.149*	0.016

*Burkina Faso HIV Survey (youth, aged 15-24)*										

% exposed to any of 3 ads	550^d^	51	13	37	28	48	81	<0.001**	0.236*	0.027

* p < 0.05, ** p < 0.01

a: Respondents were pregnant women or caregivers of children under five in sampled households

b: Respondents were all non-pregnant women surveyed who had ever heard of an IUD

c. Respondents were all non-pregnant women surveyed

d: Respondents were all youth surveyed who had seen any ad on HIV in the past two years

In the Nepal malaria prevention program, the wealthiest households in the sample received a higher concentration of exposure to BCC messages, with 50% of the households in the lowest quintile having seen a PSI-branded poster, while 78% of those in the richest quintile saw it (concentration index of 0.085) (Table 3). The proportion of households that received an LLIN home visit was not significantly different from one quintile to another (χ^2 ^= 0.221); however, the concentration index (0.056) did indicate a significant difference from zero (p = 0.019).

The exposure variables for the Nepal family planning survey also showed that the wealthier were exposed to the BCC messages more than the poorer populations (Table 3). We calculated concentration indices for exposure to any IUD message and to the branded IUD advertisements; results were 0.096 (p < 0.01) and 0.149 (p < 0.01), respectively. The branded IUD campaign exposure also generated a fairly steep gradient, with the wealth quintiles ranging from 35% in Q1 to 71% in Q5 (χ^2 ^<0.001).

### Three-way analysis: health outcomes by intervention exposure and equity

In addition to assessing the two-dimensional relationships between wealth and health outcomes, or wealth and exposure, we considered a three-way analysis of outcome by exposure and wealth in Table 4 presenting concentration indices for each outcome by exposure group. We also show one graphical example in Figure 7, chosen for illustrative purposes. In the Nepal malaria survey, the proportion of children under five who slept under an LLIN was skewed to the poor, with negative and significant concentration indices for both exposed and unexposed groups (Table 4). Exposure to a PSI-branded poster was correlated with lower inequity in the use of an LLIN among children under five (p < 0.001).

**Table 4:** Health outcomes and concentration indices by exposure to PSI interventions

Outcome	Exposure	Proportion Exposed	Concentration Index_exposed_ (n, SE)	Concentration Index_unexposed_ (n, SE)	P-value (H_0_: C. Index_exposed _= C. Index_unexposed_)
*Nepal Malaria Survey*					

Children under 5 under any bednet	Saw PSI-branded poster/leaflet	67%	0.002 (1201, 0.003)	0.014 (604, 0.010)	p = 0.146

Pregnant under any bednet	Saw PSI-branded poster/leaflet	79%	0.008 (147, 0.010)	0.149* (47, 0.053)	p < 0.001*

Children under 5 under LLIN	Saw PSI-branded poster/leaflet	67%	-0.049* (1201, 0.008)	-0.129* (604, 0.029)	p < 0.001*

Pregnant under LLIN	Saw PSI-branded poster/leaflet	79%	-0.017 (147, 0.018)	0.126 (47, 0.108)	p = 0.038*

Children under 5 under any bednet	Received a home visit regarding LLIN	30%	0.008* (548, 0.004)	0.012* (1257, 0.006)	p = 0.673

Pregnant under any bednet	Received a home visit regarding LLIN	36%	na (65,na)	0.062* (129,0.021)	na

Children under 5 under LLIN	Received a home visit regarding LLIN	30%	-0.022* (548, 0.009)	-0.051* (1257, 0.014)	p = 0.188

Pregnant under LLIN	Received a home visit regarding LLIN	36%	-0.015 (65, 0.011)	0.038 (129, 0.037)	p = 0.316

*Nepal Family Planning Survey*					

Modern contraceptive use	Heard any IUD health message	41%	0.015 (332, 0.015)	0.004 (486, 0.020)	p = 0.686

Modern contraceptive use	Saw PSI-branded poster or leaflet	54%	0.006 (555, 0.015)	0.071* (481, 0.026)	p = 0.026*

*Burkina Faso HIV Survey (youth, aged 15-24)*					

Condom at last sex with regular partner	Saw any ad	52%	0.053 (265, 0.030)	0.140* (246, 0.060)	p = 0.186

Consistent condom use with regular partner	Saw any ad	52%	0.080 (265, 0.038)	0.176* (246, 0.071)	p = 0.224

Condom use at last sex with occasional partner	Saw any ad	42%	0.170* (44, 0.064)	0.023 (62, 0.093)	p = 0.235

Consistent condom use with occasional partner	Saw any ad	42%	0.223* (44, 0.101)	0.089 (62, 0.187)	p = 0.575

*Concentration indices are statistically significantly different from zero at p < 0.05.

In the Nepal family planning survey, those exposed to the branded IUD poster or leaflet had a more equitable distribution of modern contraceptive use, compared to those in the unexposed group. The difference between the exposed and unexposed groups was statistically significant (p = 0.026). A graphical comparison of the outcome by wealth quintile and exposure depicts the proportions of women, by wealth quintile, using modern contraceptives. As seen in Figure 7, there was a relationship between exposure to the PSI-branded IUD poster or leaflet and modern contraceptive use across each wealth quintile.

In the Burkina Faso survey, condom use outcomes were more equitably distributed among the exposed group compared to the unexposed group for those with regular partners. For those reporting use with occasional partners, the unexposed group had a more equitable distribution of outcomes. However, concentration indices for the exposed and unexposed were not significantly different from each other for either condom use outcome.

## Discussion

This study compares two measures of equity, concentration indices and wealth quintiles, and considers the benefit of using both measures together to inform programmatic decision making, benchmarking these calculations against a national reference population. Having reliable and actionable measures of health equity is especially important for social marketers and other implementers in global health who target the poor. Ultimately, implementers need to be able to assess a program's capability of reaching the poor, and to demonstrate progress over time.

We calculated wealth quintiles and concentration indices to measure the distribution of health outcomes by wealth and to assess whether interventions had reached the poor. Our SES measure was benchmarked against a national reference population, improving generalizability. Each measure enables different aspects of programmatic decision making and makes up for shortcomings of the other measure. For example, concentration indices provide a summary estimate of equity with statistical significance while stratification by quintile makes it easier for implementers to see where better targeting is needed to reach the poor. For additional advantages of this combined approach, see Table 5.

**Table 5:** Benefits of combined approach for measuring equity in health outcomes and intervention exposure

Equity Metric	Disadvantage of Using Method Alone	Benefit of Combined Method
**Concentration Index**	Challenging to assess programmatic significance of a statistically significant concentration index	Threshold still unknown, but more data provided to understand programmatic significance, so equity and progress towards program goals can be measured simultaneously
	
	Cannot detect non-linear outcomes	Graphical analyses of quintiles show non-linear differences in outcomes
	
	Does not indicate which proportion of population outcome or if outcome is high or low	Wealth quintile graphs show levels of outcome in population
	
	Does not indicate how the wealth distribution of the sample compares with the national population	Use of standard asset list and DHS data as reference population shows wealth distribution relative to broader population

**Proportion by Wealth Quintile**	Challenging to do longitudinal, multi-country, or multi-outcome comparisons	Concentration index as a summary number enables statistical comparisons between multiple datasets
	
	Does not give a conclusive determination of equity	Provides a numerical estimate of equity with statistical significance, with the stratification by wealth quintile providing a comprehensive and nuanced equity assessment of the outcome measure

Using this combined approach, we are able to make a summary health equity assessment of PSI program achievements from the surveys analyzed in this study. Findings from the Nepal malaria survey showed that wealth quintiles and concentration indices operated in the same direction, suggesting that use of any bednet was concentrated among children of wealthier households, while children in poorer households were more likely to sleep under an LLIN, the actual target of the program. Patterns for both bednet outcomes suggested that wealthier pregnant women slept under bednets more frequently, but the small sample of pregnant women made it difficult to identify statistically significant trends. Exposure to the PSI program, either through viewing a PSI-branded communication or receiving a home visit, tended to favor wealthier households. However, the three-way analysis of concentration indices of bednet use by exposure group provided evidence that the program succeeded in targeting poorer households with PSI-branded posters or leaflets. This analysis also showed the program contributed to a correlation between wealth and LLIN use that favored the poor.

For our family planning example, also from Nepal, we were able to compare equity in modern contraceptive use from the PSI survey to the nationally representative DHS. Although both surveys had comparable concentration indices, presentation of wealth quintiles showed that levels of contraceptive use were higher overall and that contraceptive use was not linearly distributed in the PSI survey, with Q2and Q3 comparatively disadvantaged. This approach could be used in other settings to track program progress against national trends, although it would require comparable measurement across the two data sources. Evidence specific to the PSI family planning survey suggested that messages on IUDs were more likely to reach wealthier women, and exposure to the PSI-branded IUD communications was particularly inequitable, with exposure concentrated in Q5. However, calculating separate concentration indices for modern contraceptive use by exposure status showed that the program likely contributed to more equitable contraceptive use among women exposed to the program.

Evidence from Burkina Faso told another story. In this setting, condom use with regular partners was low overall, but more common amongst the wealthy. Condom use with occasional partners was generally concentrated amongst the wealthy, but wealth quintiles were particularly important for demonstrating that concentration amongst the wealthy was driven by high use in Q5, with generally low consistent condom use in less wealthy quintiles. The concentration index for intervention exposure showed high inequality, with very low coverage in Q1 that rose quickly to relatively similar levels for the three middle quintiles. Exposure coverage was much higher in Q5. Analysis of concentration indices for condom use outcomes by program exposure did not yield any consistent evidence that the program may have contributed to greater equity in condom use with either regular or occasional partners. We can conclude that the program was likely operating in an environment with inequitable use of condoms and that more careful targeting of program strategies to reach the poor would be merited.

This combined approach of analyzing wealth quintiles and the concentration index provided evidence that helps pinpoint which socioeconomic strata benefit more from the intervention, helping implementers know whom to target when designing new interventions or adjusting existing ones. In general, trends in wealth quintiles and concentration indices were comparable in the data we examined, but the ability of wealth quintiles to show non-linear outcomes graphically provides greater nuance in understanding exactly how these measures were concentrated. To answer the question of whether programs are actually reaching the poor, nuanced insights for program targeting can also be derived from our analysis of equity in exposure to interventions. Positive and significant concentration indices from all three surveys suggested a positive relationship between wealth and media exposure that could require implementers to consider their outreach strategies.

When making decisions about the social marketing intervention, it is also important to not dismiss data that may still be useful, even if potential confounders may be present in one analysis of the variables. For example, while some may argue that the relationship between intervention exposure and wealth may be confounded by education, it does not alter the programmatic conclusions drawn from understanding who has been exposed to an intervention. Both education and an asset index-based wealth index are proxy measurements for socioeconomic status, allowing the program implementer to learn more specific details about the socioeconomic groups reached by the intervention.

We also examined the relationship between equity in intervention exposure and outcome to further support decision making on the equity implications of program strategies. Figure 4 presented the relationship between SES and modern contraceptive use, showing that the wealthy were advantaged in using modern contraceptives in Nepal. Further analysis shown in Figure 7 demonstrated how this relationship may be influenced by exposure to IUD messages as there was greater use of modern contraceptives among those exposed to a PSI-branded IUD message. A cautious interpretation of these data would note that exposure to a PSI message is correlated with improved equity even though modern contraceptive use was inequitably distributed. This information could be used in targeting and tracked over time.

The combined approach we have used corresponds to standard methods used by the World Bank to measure equity and financial protection in the health sector, and the presentation of wealth quintiles and concentration indices is in line with other health equity studies [29-31]. Our approach expands on these methods in several key areas, however. With our aim of assessing health in social marketing programs, we worked with sub-national datasets from defined geographic areas that reflect programmatic implementation plans. We are therefore limited in being able to make national policy recommendations that other health equity studies have had [7,8,31]. Second, in light of our concern with programmatic decision making, our calculation of the asset index for determining wealth quintiles and concentration indices differed from the nationally representative populations used by the World Bank and others [32,33]. We instead benchmarked against a national reference population in order to make the equity estimates from a program area meaningful and comparable. Further, we considered health equity in several health outcomes and in measures of intervention exposure that tend to occur outside of the mechanism of health care delivery. This approach corresponds with our interest in providing programmatically meaningful evidence for social marketing interventions that operate via health promotion and behavior change communications strategies which often do not intersect with health care delivery.

As discussed in Table 5, combining these two methods helps make the results easier to interpret, and therefore, use for programmatic decision making than if the concentration index is used on its own. Using two pieces of evidence (wealth quintiles plus concentration index) enables implementers to address apparent inequities by designing action-oriented strategies. At the same time, this combined approach also makes it possible to make summary assessments of programmatic inequities across interventions, countries, and time to assist in strategic decision making at a programmatic and an institutional level.

### Programmatic limitations of approach

While this method for measuring health equity in interventions offers notable benefits, the approach has limitations. First, the proposed use of DHS or other population-level surveys as reference population data is not applicable to all of the places where social marketing organizations implement their initiatives. For example, among the countries where PSI operates, procurement of national wealth data is difficult in China, Myanmar, Papua New Guinea, Somaliland, and South Sudan. In these cases, assessment of health equity in programs, and comparison to their respective national contexts, will only be possible if the PSI surveys are nationally representative or another data source becomes publicly available. Assessment of health equity in programs within the target population is still feasible, however.

A second limitation concerns the measurement of health equity in programs among target audiences that are mobile or not living in households. This issue is particularly salient for HIV prevention programs. Among these populations, it may be challenging, if not impossible, to create an asset list, even though factor weights for a standard list may be available from DHS data. As a result, researchers will face difficulties creating a reliable measure of wealth for these groups, and may need to use a proxy measure of SES, such as education, which is easier to obtain. For example, the transgendered population of Thailand is highly mobile, traveling between tourist areas, urban centers, and/or their native homes at different times of the year [34]. Depending on when and where HIV prevention program surveys are implemented, as well as their sampling mechanisms, researchers may be unable to gather data on the household assets of this group, or may choose to assess relative wealth based on income.

A third challenge of our new approach is the task of discerning when inequity in the results should be acted upon. While the graphical analysis of indicators by wealth quintiles may provide greater nuance than concentration indices, and therefore, greater ease in understanding when results are of programmatic concern, our combined approach does not provide guidelines or threshold numbers. Given the multitude of factors affecting social marketing programs that measure health equity and the unique contexts of each one, it may be difficult to develop a set of guidelines, quantifiable or not, that can be universally applied.

## Conclusion

Understanding the relative equity of an intervention's target audience, not to mention a program's exposure and outcomes, is essential for many social marketing agencies and organizations applying a total market approach, a strategic framework that donors increasingly expect these organizations to adopt [12]. With this approach, social marketers simultaneously seek both health impact and market growth, in order to promote long-term access, availability, use, and ultimately, impact of the promoted health product or service. To successfully expand the market, these organizations need to ensure that intervention strategies encompass the different segments of the market - public, socially marketed, or commercial - and appropriately target the individuals they serve, based on socioeconomic status. Individuals need to have access to products and services at prices they can afford, access that can be reduced for the poor and vulnerable if wealthier people purchase heavily subsidized products designed for those of lower economic status [14]. Equity monitoring, therefore, is paramount to ensuring that intervention design is sound and program objectives are achieved.

To do so effectively and to make any needed adjustments to intervention strategies, health equity assessment tools must provide adequate details about an intervention, including the nuances of which SES segments are reached by program messages as well as when equity results are actionable. Until now, social marketers have not had such a methodology available. The combined approach of wealth quintiles and concentration indices introduced in this study fills this gap, ensuring that global health social marketing organizations can further increase access to the health products and services needed by the communities they serve.

## List of abbreviations

DHS: Demographic and Health Surveys; PSI: Population Services International; LMIC: low- and middle-income country; MDG: Millennium Development Goal; TMA: total market approach; SES: socioeconomic status; PCA: principal components analysis; BCC: behavior change communications; HIV: human immunodeficiency virus; LLIN: long-lasting, insecticide-treated bednet; IUD: intrauterine device; PPS: probability proportional to size.

## Competing interests

The authors declare that they have no competing interests.

## Authors' contributions

NC and RF conceived of the study. NC and RF designed the study methodology, and NB conducted statistical analyses. All authors participated in writing the first draft and revisions of this paper. All authors read and approved the final manuscript.

## Supplementary Material

Additional file 1 **Asset ownership (%) by study population**. These data show the proportion of each study population (PSI and DHS) who owned the assets that were used in the calculation of the asset index. Click here for file

Additional file 2 **Health outcome by quintile in PSI surveys**. This file contains additional data on the proportion of the study population attaining each health outcome, by quintile. These are the same data represented in Figures 2 through 6. Click here for file

## Declarations

This article is part of the supplement of *BMC Public Health *Volume 13, Supplement 2, 2013: Use of health impact metrics for programmatic decision making in global health. Population Services International, a registered non-profit organization, provided the funding for the publication of this supplement. The full contents of the supplement are available online at http://www.biomedcentral.com/bmcpublichealth/supplements/13/S2.
