# Supplementary material for: Equity monitoring for social marketing: use of wealth quintiles and the concentration index for decision making in HIV prevention, family planning, and malaria programs
Source: BMC Public Health. 2013 Jun 17;13(Suppl 2):S6. doi: 10.1186/1471-2458-13-S2-S6 (PMC3684531; doi:10.1186/1471-2458-13-S2-S6)
Supplement: Additional file 1 — Asset ownership (%) by study population. These data show the proportion of each study population (PSI and DHS) who owned the assets that were used in the calculation of the asset index. [file 1471-2458-13-S2-S6-S1.PDF]

Additional file 1. Asset ownership (%) by survey population

Asset	Nepal DHS 2010 (n=10,826)	Nepal Malaria 2010 (n=3327)	Nepal FP 2010 (n=1078)	Burkina Faso DHS 2003 (n=9097)	Burkina Faso HIV 2011 (n=730)
Electricity	77	83	91	12	26
Radio	51	64	63	64	90
TV	46	51	65	12	35
Refrigerator	11	7	16	5	8
Bicycle	33	64	62	77	92
Motorscooter	11	9	17	25	56
Mobile phone	74	67	89	3*	na
Non-mobile phone	11	9	13	na	na
Animal cart	2	9	6	na	na
Table	52	60	67	na	na
Chair	45	66	68	na	na
Bed	90	96	97	na	na
Sofa	14	14	21	na	na
Cupboard	42	50	58	na	na
Clock	44	78	74	na	na
Fan	36	55	59	na	na
Car/Truck	2	2	3	2	2
Improved water source	86	91	93	na	na
Running water	na	na	na	23	21
Improved toilet	91	60	68	23	7
Improved cooking fuel	26	18	34	4*	na
Flooring – natural	64	72	56	58	42
Flooring – rudimentary	2	3	1	<1	<1
Flooring – finished	34	25	43	42	57
Roof – natural	17	12	19	na	na
Roof – rudimentary	2	1	0	na	na
Roof – finished	81	87	81	na	na
Walls – natural	8	11	11	na	65*
Walls – rudimentary	49	48	37	na	<1*
Walls – finished	43	39	52	na	35*

Notes: “na” not available, *not used in asset calculations
