# Supplementary material for: Equity monitoring for social marketing: use of wealth quintiles and the concentration index for decision making in HIV prevention, family planning, and malaria programs
Source: BMC Public Health. 2013 Jun 17;13(Suppl 2):S6. doi: 10.1186/1471-2458-13-S2-S6 (PMC3684531; doi:10.1186/1471-2458-13-S2-S6)
Supplement: Additional file 2 — Health outcome by quintile in PSI surveys. This file contains additional data on the proportion of the study population attaining each health outcome, by quintile. These are the same data represented in Figures 2 through 6. [file 1471-2458-13-S2-S6-S2.PDF]

Additional file 2. Health outcome by wealth quintile in PSI surveys

Outcome	N	Overall	Q1	Q2	Q3	Q4	Q5	χ^2	C. Index	$SE_{C.Index}$
Nepal Malaria Survey										
% of children under 5 who slept under any bednet the previous night	1805	92	90	89	93	93	95	0.067	0.012*	0.004
% of children under 5 who slept under an LLIN the previous night	1805	66	76	68	63	65	59	0.006	-0.035*	0.010
% of pregnant women who slept under any bednet the previous night	195	91	66	97	90	95	98	0.002	0.044*	0.013
% of pregnant women who slept under an LLIN the previous night	195	77	66	87	69	78	88	0.191	0.031	0.023
Nepal Family Planning Survey										
% of non-pregnant married women using modern contraceptives	1036	63	61	53	57	68	69	0.001	0.056*	0.014
Burkina Faso HIV Survey (youth, aged 15-24)										
% condom use at last sex with regular partner	523	47	13	40	30	59	54	0.000	0.137*	0.030
% consistent condom use with regular partner	523	35	13	26	20	43	45	0.000	0.182*	0.036
% condom use at last sex with occasional partner	110	61	20	50	65	56	90	0.010	0.128*	0.062
% consistent condom use with occasional partner	110	42	0	38	30	34	81	0.001	0.196	0.108

**Concentration index is statistically significantly different from zero at p<0.05.*
